# A Plea for More Frequent Employment

**Published:** 1919-11-01

**Authors:** 


### LUMBAR PUNCTURE AND LATE SYPHILIS.

### A Plea for More Frequent Employment.

There is need perhaps at the outset of this note, in which we would urge the more frequent use of lumbar puncture in the diagnostic care of many cases of syphilis or possible syphilis of the central nervous system as met in private practice, to point out that this small operation is not now considered dangerous, it is certainly relatively easy to perform, and it is generally held to be perfectly satisfactory, first mildly to prepare the patient, afterwards to keep him quiet in bed for twenty-four hours, and to allow him up next day, except in the case of puncture headache, which though sometimes alarming calls merely for rest and general measures.

### The Wassermann Test.

On every hand by the increased use of the Wassermann test we see a great number of cases of syphilis newly diagnosed, in which the infection occurred years before, and the good results attending the prompt attack on the surviving spirochætes which are assumed to be present is a justification in itself. Perhaps not so invariably and startingly successful, the corresponding treatment in the case of syphilis of the C.N.S., is, nevertheless, both definitely indicated, and should be started the moment, a positive diagnosis is made.

### Recent Practice.

Details of such treatment itself space will not allow us to discuss, but the urgency is in any event in diagnosis, and the great question thence arising is whether a careful history and physical examination will reveal spirochætal activity in the nervous system or should the special diagnostic aid of the lumbar puncture be sought. The clinical expert will doubtless say that the latter is a useful confirmatory test, but not essential. Nevertheless, relatively few of us can claim to be such experts, and it is noticeable that in the Services, and more recently in the instance of Ministry of Pensions investigations, the laboratory test is, wherever possible, applied to the cerebro-spinal fluid. As a result, the reaction and puncture have been applied to an enormous number of cases in which there was any possibility whatever of a "specific" cause, and from all evidence we can obtain it seems undoubted that this procedure has revealed a number of positive instances in which there were practically no other signs or symptoms of venereal infection at all, to the average medical observer at least. Also in a number of cases, granted the old venereal infection, there were no present signs, but still the cerebro-spinal fluid gave a positive W.R., from which, presumably, a future central nervous system manifestation of the disease might be prognosed.

### Missed Cases.

Authorities working under hospital conditions variously place the number of such cases as between 2 and 5 per cent., in which neither history nor physical examination called the attention of the observer to the central nervous system, even after syphilis was known to be present. Were the puncture not performed these cases would be almost invariably missed, and that under ideal opportunities for examination. How much more easy is it for the same cases to slip by under the less perfect medical surroundings of private general practice? Critics there may be of the value attaching to any known treatment for these syphilitic nervous cases, but the fact remains that we may never know of the existence of at least some of their number if resort be not made to the relatively simple procedure of lumbar puncture.

